# A Case Study Describing a Community-Engaged Approach for Evaluating Polycyclic Aromatic Hydrocarbon Exposure in a Native American Community

**DOI:** 10.3390/ijerph16030327

**Published:** 2019-01-24

**Authors:** Diana Rohlman, Jamie Donatuto, Myk Heidt, Michael Barton, Larry Campbell, Kim A. Anderson, Molly L. Kile

**Affiliations:** 1 School of Biological and Population Health Sciences, College of Public Health and Human Sciences, Corvallis, OR 97331, USA; diana.rohlman@oregonstate.edu; 2 Swinomish Indian Tribal Community, La Conner, WA 98257, USA; jdonatuto@swinomish.nsn.us (J.D.); mheidt@swinomish.nsn.us (M.H.); lcampbell@swinomish.nsn.us (L.C.); 3 Research Translation Core, Superfund Research Program, Oregon State University, Corvallis OR 97331, USA; Michael.Barton@oregonstate.edu; 4 Department of Environmental and Molecular Toxicology, Oregon State University, Corvallis, OR 97331, USA; im.anderson@oregonstate.edu

**Keywords:** community based participatory research, passive sampling, PAHs, environmental pollutants, air pollution, air toxics, air quality, silicone, Tribal-University partnership, environmental health education

## Abstract

In 2015, the Swinomish Indian Tribal Community (SITC) was impacted by an air toxic release from one of two nearby oil refineries. This experience motivated SITC members to learn more about their exposure to air toxics. On the invitation of SITC, this community-based study measured personal exposure to polycyclic aromatic hydrocarbons (PAHs) and conducted interviews with the volunteers to evaluate perceptions of the data and experience of participating. Non-smoking SITC members were recruited in March 2016 (*N* = 10) and January 2017 (*N* = 22) with seven volunteers participating both times. Volunteers wore a wristband passive sampler for 7 days and completed daily activity diaries. Wristbands were analyzed for 62 PAHs using gas chromatography mass spectrometry. Wilcoxon exact tests determined if the sum total PAHs (ΣPAH) differed by activity, proximity to the refineries, and time. Aggregated results were shared during community meetings, and volunteers received individual reports. Volunteers (*N* = 9) participated in individual interviews. All volunteers were exposed to different amounts and types of PAHs. Burning candles or using a wood stove and/or propane heating were associated with higher ΣPAH exposures. While ΣPAH was similar in both sampling periods, the composition of PAHs differed. More priority listed PAHs were detected in January (*N* = 17) versus March (*N* = 10). Among volunteers who participated in both sampling events, exposure to four PAHs significantly differed between seasons. Overall, volunteers reported that the study made them more aware of air pollution sources in their community. They also commented that the chemical nomenclature was difficult to understand, but appreciated the individual reports that allowed them to visually compare their data to the distribution of data collected in their community. For volunteers with lower exposures, these comparisons gave them relief. However, volunteers with higher exposures reported concern and several changed their behaviors to reduce their exposure to known PAH sources. This study provided an opportunity for SITC members to learn about their personal exposure to a class of air toxics within the context of their community. While the limitations of the study hindered the ability to identify sources of air toxics in the community, this activity appeared to raise awareness about ambient and indoor air pollution among the volunteers.

## 1. Introduction

In the Puget Sound of Washington State, there are five major oil refineries, all of which are located on or near Indian reservations. These oil refinery complexes produce a host of petroleum products, including gasoline, fuel oil, diesel fuel, butane, propane, and petroleum coke. Two of these refineries are located near the Swinomish Indian Tribal Community (SITC). When one of the oil refineries did not decontaminate an industrial flare before shutting down for a scheduled routine maintenance in February 2015, there was an uncontrolled release of air toxics [1,2,3]. The resulting poor air quality affected people living near the refineries who reported noxious odors, burning eyes, irritated throats and lungs, and headaches [4]. The Northwest Clean Air Agency received 67 complaints, and SITC reported 176 written accounts of people who had been affected by the poor air quality with several Tribal members reporting that they sought medical care [5]. One of the concerns expressed by SITC during, and after, the industrial incident was the lack of communication by the oil refinery or regulatory agencies regarding the types of chemicals to which local area residents were exposed. In was not until April 2015 that the oil refinery publicly released information stating that the chemicals that were unintentionally emitted were hydrogen sulfide, mercaptans, benzene, hydrocarbons and dimethyl sulfide [1,2,3,4,5].

Within a month of the toxic air release, the SITC Senate prioritized additional community air toxics monitoring. They wanted data that would complement the existing air monitoring conducted by the SITC Air Quality Program, which monitors ozone, sulfur dioxide, and nitrogen dioxide, and meteorological conditions including wind speed and direction, temperature and relative humidity, barometric pressure, solar radiation and precipitation. These data are uploaded into the Environmental Protection Agency’s Air Quality System repository [6]. The SITC Senate was supportive of additional environmental monitoring that could measure air toxics in their community. They were specifically interested in data that could be used to evaluate the impact of the oil refineries, the emissions from diesel locomotives that bring more than 100 cars to the refineries each day that run across the northern part of the Swinomish Reservation, and other industrial sources within the SITC air shed. The SITC Senate was also supportive of citizen science activities that could allow Tribal members to conduct personal and community air quality monitoring. Upon learning of the community’s experience and interests, the Oregon State University Superfund Research Program’s Community Engagement Core (CEC) contacted SITC. One of the CEC’s primary goals is to reduce environmental disparities experienced by Pacific Northwest Tribes by developing collaborative community-engaged research between Oregon State University Superfund Research Program scientists and Tribes. The SITC invited the CEC to collaborate on air toxic monitoring projects that included using silicone passive sampling wristbands to measure personal chemical exposures [7,8,9]. This exposure assessment method is non-invasive, simple to use, and provides individual time-averaged exposure data [10,11,12,13].

Of the different chemical classes that were in the refinery air toxic release, polycyclic aromatic hydrocarbons (PAHs) were identified as chemicals of interest that can be measured with the silicone passive sampling wristbands [7,14,15]. There are many different types of PAHs that are produced by natural and anthropogenic sources when organic materials are burned [16]. Importantly, crude oils contain 0.2% to 7% PAHs with configurations ranging from two to six rings [17], and PAHs can be generated by refining petroleum products, leading to environmental and occupational exposures [18,19,20]. Diesel locomotives are also a known non-point source of PAH emissions [21]. Additionally, PAHs are produced by other sources including tobacco smoke [22], gas or wood burning stoves or fireplaces [23], burning candles or incense [24], vehicle and engine exhaust [25], wildfires or vegetative burning [26] and asphalt or tar [27]. Subsequently, PAHs are ubiquitous environmental contaminants in the ambient and indoor environment [28,29]. In 1976, the United States Environmental Protection Agency listed 16 PAHs (e.g., the “priority list”) that are commonly found in the environment to estimate the carcinogenic risk of PAH-related air pollution [30]. However, this list has subsequently been expanded to 26 PAHs [31] and contains several probable human carcinogens (e.g., benzo[*a*]anthracene, chrysene, benzo[*a*]pyrene, benzo[*b*]fluoranthene, benzo[*k*]fluoranthene, dibenz[*a,h*]anthracene, and indeno[1,2,3-*c,d*]pyrene) [32,33,34,35]. Several PAHs including anthracene, benzo[*a*]pyrene, and naphthalene are also known to cause irritation, inflammation, dizziness, vomiting, and headaches [36,37,38]. PAHs are also known to enhance allergic inflammation [39], increase incidence of childhood asthma [40,41,42,43,44], cardiovascular disease [45,46], and adverse neurodevelopment [47,48]. 

Together, the SITC and CEC designed a community-based participatory research project that focused on measuring personal exposures to PAHs following the research framework prescribed by the National Institute of Environmental Health Science for ensuring community engagement [49,50]. The objectives of the study were three-fold: (i) perform preliminary data collection on ambient PAH personal exposures and; (ii) utilize data to inform decision-making and increase environmental literacy around air pollution and risk reduction strategies and; (iii) evaluate the study for changes in knowledge related to air pollution. The decision was also made to recruit SITC Reservation residents who are American Indian/Alaska Native (enrolled with SITC or another tribe). Recruited community members could volunteer to wear a silicone wristband for 7 days to learn about their personal exposure to PAHs, and contribute to providing data on PAH exposures in their community. SITC Tribal members could volunteer to wear a silicone wristband for 7 days to learn about their personal exposure to PAHs, and contribute to providing data on PAH exposures in their community. Since the SITC Reservation often experiences temperature inversions, it was decided to have two sampling periods (Spring and Winter). Following each sampling period, individual data were returned to study participants and aggregate data were reported back to SITC governance and the community. This approach is consistent with incorporating community feedback on the study approach, design, and translation efforts [51,52,53,54]. Additionally, research has shown that returning data to study participants is ethical, increases environmental literacy, and empowers communities to take action to reduce their exposures even when health effects or regulatory standards do not exist [54,55,56,57,58,59]. Therefore, we worked closely with researchers, institutional review boards, and our community partners to navigate the intricacies of returning non-clinical PAH data captured by the passive sampling wristbands used in this study. Finally, we conducted individual interviews with volunteers to learn their opinion regarding the utility of this study and to evaluate the success of the approach used for data dissemination.

## 2. Methods

### 2.1. Study Location and Population

**Figure 1:**
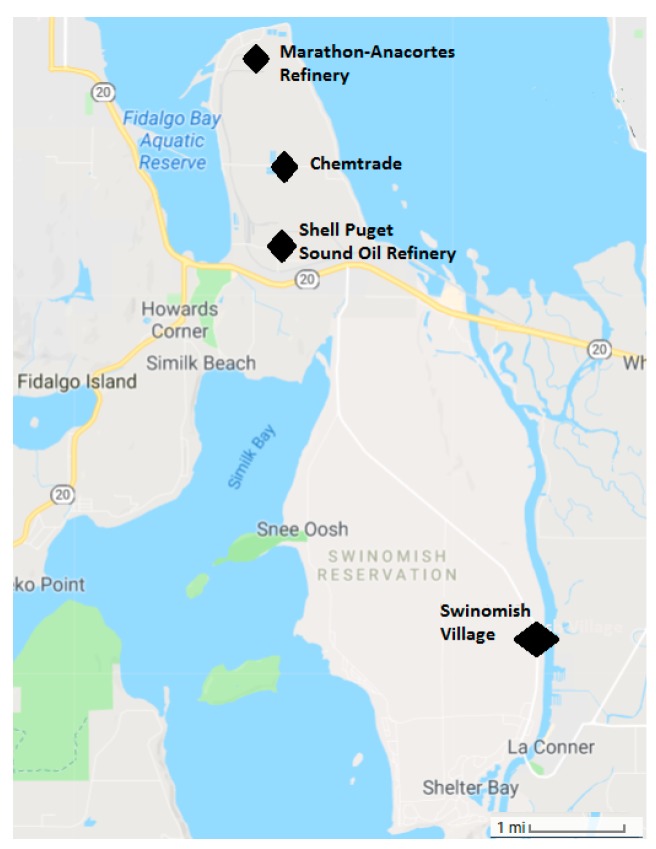
Map of the local area illustrating the location of the Swinomish Village and Reservation, and the oil refinery complexes.

The Swinomish Indian Tribal Community (SITC) is federally recognized Indian Tribe organized under Section 16 of the Indian Reorganization Act and governed by the Swinomish Indian Senate (Senate), comprised of 11 elected representatives. Today there are nearly 1000 enrolled members. The Swinomish are one of several Indigenous peoples of the Salish Sea region known as the Coast Salish people. The Swinomish Reservation is located on the peninsula at the southeastern end of Fidalgo Island. The Reservation was established by Article 2 of the Treaty with the Dwamish Suquamish, Etc., 12 Stat. 927 (1855). As established by the Treaty, the Reservation includes approximately 10,800 acres of upland areas and approximately 4500 acres of wet and filled tidelands; most of the Reservation is ringed by salt water. Two of the largest oil refineries in the region are within 10 miles of the Swinomish Village, where the majority of community members reside. Diesel locomotives service the oil refineries on a daily basis (Figure 1). The railroad tracks pass across the northern part of the Reservation. 

Volunteers were eligible for the study if they were 18 years or older, non-smokers, and lived on the SITC Reservation. SITC Community Environmental Health staff coordinated participant recruitment and data collection. Recruitment methods included word-of-mouth, announcements in the local community newspaper, and mailing letters of invitation to SITC members who lived on the Reservation. There were two sampling periods March 22–28, 2016 (Spring), and January 25–31, 2017 (Winter). 

In total, 10 and 22 Tribal members were recruited to participate in Spring and Winter, respectively. Of those, seven participated in both sampling periods. Following the last sampling (Winter), volunteers were re-contacted to ask if they would be willing to be interviewed in order to gain their perspectives on the study. Ultimately, nine volunteers were interviewed by telephone using a semi-structured questionnaire. These interviews evaluated perceptions about exposure to PAHs, questions about individual behaviors after participating in the study, perceptions around the usefulness of the study for the participants and the community, and the clarity of the reports in translating data back to the individual participants. Upon the request of the community, interviews were recorded by taking notes during the interview. The transcript notes were then coded using a deductive approach. 

### 2.2. Ethical Statement

All study activities received IRB approval from Oregon State University (IRB number: 7164) and the Northwest Indian College (IRB project number: # 2016-001). Volunteers provided written consent prior to participating in any study activities. As a small token of appreciation for their time, participants received a $50 gift card per sampling event, and lunch was provided to anyone who attended the community meeting where aggregated results were shared with the volunteers and the community. Additionally, all study activities occurred under a Data and Material Sharing and Ownership Agreement (DMSOA), which is an additional layer of protection to Institutional Review Board protections. The DMSOA stipulates that all data and materials used or generated during the project belong to SITC and that SITC controls the use of the data and materials, and dissemination of information [60]. Furthermore, the review process was outlined in advance and included a Tribal Advisory Committee (TAC) who reviewed all results and documents prior to dissemination and distribution. All final documents were then sent to the SITC Health, Education and Social Services (HESS) committee, which included Swinomish Senate and Legal representatives, for input and review. 

### 2.3. Ambient Temperature

Temperature data were obtained from Weather Underground (wunderground.com), using the weather station at Skagit Regional Airport (Burlington, WA, USA), located ~6.5 miles from the center of the Swinomish Village. The average temperature in Spring was significantly warmer than the Winter (8.2 °C, range 1.7–15 °C versus 4.8 °C, range 0–10 °C, *p* = 0.0005 student’s *t*-test). However, no temperature inversion occurred during the Winter sampling time.

### 2.4. Exposure Assessment

Participants wore passive sampling silicone wristbands for 7 consecutive days during each sampling period. Afterwards, the wristband was sealed in an individual Teflon bag and shipped to Oregon State University for analysis. Methods describing the preparation and analysis of the wristbands have been previously published [7,10,11]. The wristband samples were quantitatively analyzed for 62 PAHs using an Agilent 7890A gas chromatograph (Santa Clara, CA, USA) interfaced with a modified Agilent 7000 GC-MS/MS following methods described in Anderson et al [11]. Instrumental limits of detection range from 0.24 to 6.44 ng per wristband. Quality control included instrument and extraction blanks and calibration verification standards for instrument performance. The concentration of PAHs in the wristbands was normalized by the mass of the average wristband (4.64 grams) which results in a unit of nanograms per wristband (ng/wristband). All detectable PAHs were summed to estimate total PAH exposure (ΣPAH), and were evaluated as single compounds.

Returned wristbands were assessed to ensure they followed protocol: (i) Worn for 7 days (±1 day); (ii) On and off dates/times recorded and; (iii) Appropriately sealed in an air-tight transport bag. 

### 2.5. Covariates

Volunteers completed a very brief demographic questionnaire to gather information about their gender and whether they lived near or worked in an occupation that would likely be a source of PAH exposure (e.g., an oil refinery, gas station, casino, roofing, road paving, or place where smoking was permitted indoors). This information was intentionally limited to preserve the anonymity of volunteers in this small community. Additionally, volunteers were asked to keep a daily activity diary while they wore the wristband that asked about frequency of contact with potential sources of PAHs—burning candles or incense, cooking practices (e.g., grilling food, or burning food), home heating sources (e.g., wood burning stove, electricity, or propane), personal care products, oil-based paints, caulks/sealants, construction materials, cleaners and solvents, moth balls, and gasoline. 

### 2.6. Statistical Analysis

Descriptive statistics were calculated for individual PAHs and for the sum of all detectable PAHs (ΣPAH). Wilcoxon exact tests (one and two-sided) were used to assess the difference in PAH exposure by sampling period, gender, proximity to known point sources, use of candles (yes/no), incense (yes/no), contact with gasoline (yes/no), exposure to burnt food (yes/no), grilling food at home (yes/no), or by home heating source (electricity only/heating that included a woodstove and/or propane). Linear regression models assessed the association between ΣPAH concentrations and the frequency of contact with specific activities during the 7 day observation period (e.g., gender (male/female), burned candles (yes/no), grilled foods (0–4 times per week vs. 5–7 times per week), primary heat type in home (electricity only, vs. other type), contact with gasoline (0–1 times per week vs. 2 or more times per week), burned incense (yes/no), burned food (yes/no), self-reported proximity to known PAH source such as oil refinery, facility that allows smoking indoors, construction activity involving tar, etc. (yes/no), and season (spring/winter). Covariates that were statistically significant at an alpha = 0.05 were included in a multiple linear regression model (e.g., burning candles, gasoline contact, and home heat). 

### 2.7. Procedures for Reporting Results

#### 2.7.1. Individual Data Reports

During enrollment, volunteers were given the option to receive their individual data, and everyone requested their individual results. These individual reports began by describing the total number of PAHs detected in the volunteer’s wristband and the absence of reporting guidance for these measurements:

“Out of the 62 PAHs we tested for, only # were found in your wristband. Only # are considered to be potentially harmful to humans. There are no regulatory guidelines or safety levels, but we have listed what information is available. While these compounds were found in your wristband, it does not mean you will experience poor health effects.”

**Figure 2:**
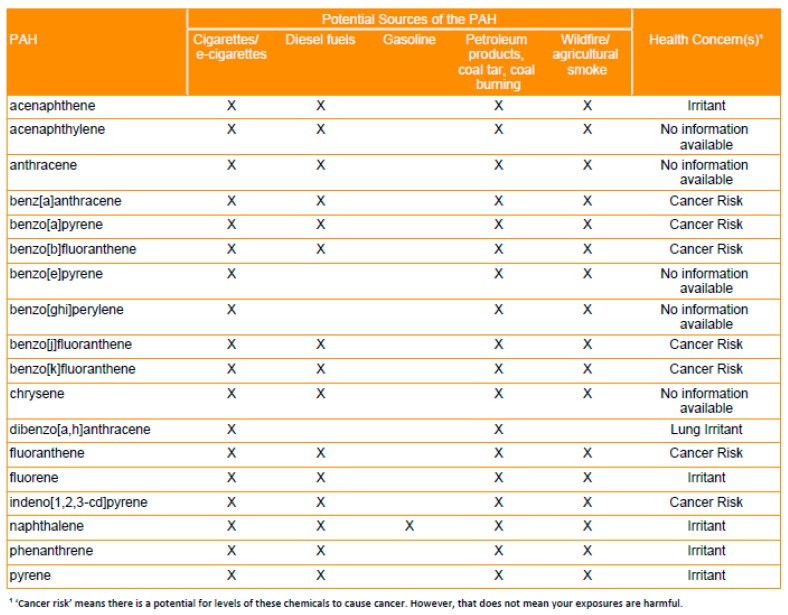
PAH (polycyclic aromatic hydrocarbon) source and health concern table for 18 priority PAHs. Priority PAHs were identified by the Environmental Protection Agency and the Agency for Toxic Substances and Disease Registry.

**Figure 3:**
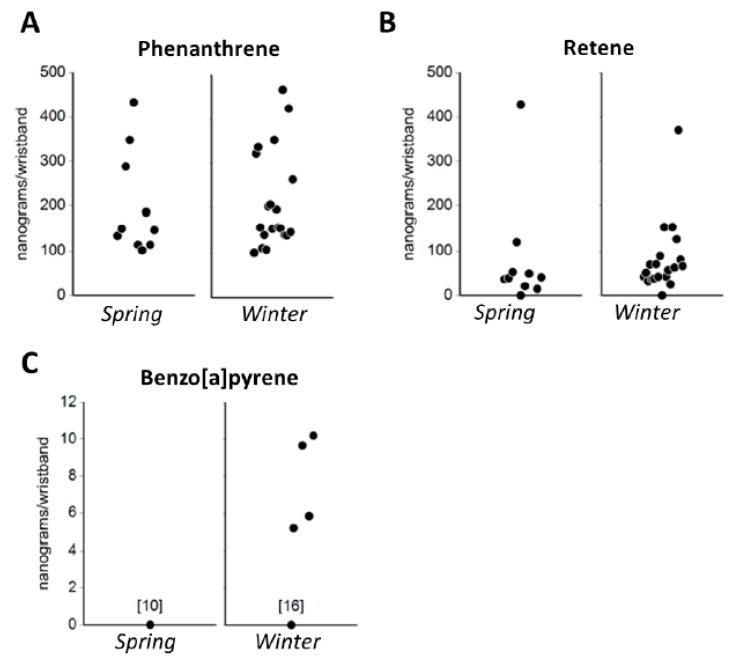
Strip charts showing the distribution of (**A**) phenanthrene, (**B**) retene, and (**C**) benzo[*a*]pyrene in the Spring and Winter deployments. These charts were used in the individual reports to place the results in the context of the study population. None of the participants in the Spring deployment had levels of benzo[*a*]pyrene at detectable levels. All units are in nanograms per wristband.

Next, a table identified which priority PAHs were detected in the individual’s wristband and indicated possible sources of exposure (e.g., cigarettes/e-cigarettes; petroleum products, coal tar, coal burning; diesel fuels; gasoline; wildfire/agricultural smoke), and potential health effects associated with these compounds (e.g., irritant includes skin or eye irritation, lung irritant, cancer risk, and no information available) (Figure 2). Health data were sourced from the United States Environmental Protection Agency, the Agency for Toxic Substance Disease Registry, and the International Agency for Research on Cancer for priority PAHs [33,61]. Then, individual data were graphically presented in strip charts that showed the individual’s data within the context of the entire sample following protocols described in Brody et al. [58]. Initially, strip charts were provided for all 62 PAHs but formative research with the TAC found that these data were overwhelming, especially given the lack of regulatory or health-based standards for these compounds. Subsequently, strip charts were prepared for three PAHs (see Figure 3) that reflected common sources and that had well characterized toxicity: (1) Phenanthrene, a lung irritant that is produced by the combustion of fossil fuels and can also be detected in tobacco and wood smoke; (2) Retene, a lung irritant that is primarily produced by the combustion of soft wood and biomass [62] and emitted from pulp and wood processing [63]; and (3) Benzo[*a*]pyrene, a Group 1 carcinogen that is produced by the combustion of fossil fuels and wood and can also be found in coal tar, tobacco smoke, smoked foods, and grains. Additionally, the data for individuals who volunteered in Spring and Winter were graphed using bar charts that allowed them to easily compare their exposures between each sampling period. 

#### 2.7.2. Aggregate Data Report

All data were aggregated to answer the community’s questions about “What are we being exposed to? Are these exposures harmful to our health? Where could these pollutants be coming from? Are we being exposed to these pollutants from the oil refinery? What can we do to reduce our exposures to these pollutants? Was there a difference between the Spring and the Winter?” For the Spring aggregated report, the primary goal was to share information about the amount of PAHs that were measured in the community and illustrate between person variability using a stacked bar chart (text and tables were used to describe the possible sources of PAH exposure and health effects associated with the six PAHs that were detected in all 10 wristbands (e.g., phenanthrene, 2-methylphenanthrene, naphthalene, 2-methylnapthalene 1.6-dimethylnapthalene 2,6-dimethylnapthalene). Simple infographics were included to illustrate strategies for reducing exposure to PAHs (e.g., ventilation when cooking or grilling, maintaining wood stoves and chimneys, choosing better candles, and avoiding e-cigarette/cigarette smoke) (Supplementary Figure S1). For the Winter report, all data were pooled for analysis and solid bar charts were used to illustrate any statistical differences in ΣPAH by gender and different activities. Seasonal differences in ΣPAH and for individual PAH compounds were evaluated using data from the seven individuals who participated in both sampling periods and were reported graphically using bar charts. For the Winter reports, a Venn diagram was also included to illustrate the number of priority PAHs detected during each sampling season (Supplementary Figure S2).

## 3. Results

This study included a convenience sample of 25 non-smoking Tribal members (Table 1). Overall, 76% of the volunteers were female and lived and/or worked near PAH sources (e.g., oil refinery, gas station, casino, roofing/road paving, or operating a woodstove). Many of the volunteers explained that they wanted to be in the study because they had been personally impacted by the oil refinery air toxic release in 2015. Others volunteered because they were curious about the chemical exposures they encounter on a daily basis. 

### 3.1. Compliance

In the Spring (*N* = 10) and Winter (*N* = 22) all volunteers returned their wristbands having worn them for 7 days. However, two Winter wristbands were not placed in their air-tight bags within the time period required from the protocol. This deviation from the protocol prevented their data from being directly comparable to the other volunteers and subsequently these two data points were excluded from the analyses. In total, 23 wristbands were used to describe PAH levels in the community and for any statistical analyses. Overall, compliance was very high, and volunteers reported having no problem with wearing the wristband passive samplers.

### 3.2. PAH Exposures

The data showed that all volunteers were exposed to PAHs. In Spring and Winter, a total of 25 and 43 PAHs were detected in the wristbands (Table 2). Across all participants and both time periods, 44 unique PAHs were detected of which 17 were priority PAHs. More priority PAHs were detected during the Winter (*N* = 17) than the Spring (*N* = 10) sampling period. 

**Figure 4:**
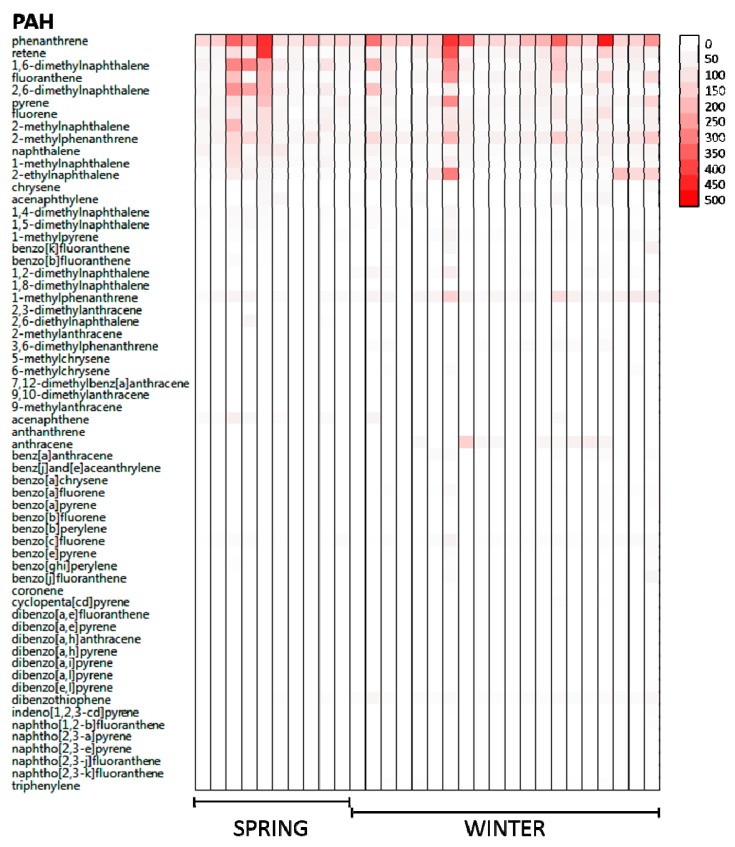
A total of 62 PAHs were analyzed in each wristband in the Spring (*n* = 10) and the Winter (*n* = 20). PAH concentrations are denoted via color gradient, ranging from not detected (white) to higher concentrations (red) in nanograms/wristband, showing distribution of PAHs across participants during the Spring and Winter deployments. Priority PAHs include: Acenaphthene, Acenaphthylene, Anthracene, Benz[*a*]anthracene, Benzo[*a*]pyrene, Benzo[*b*]fluoranthene, Benzo[*e*]pyrene, Benzo[*g,h,i*]perylene, benzo[*j*]fluoranthene, benzo[*k*]fluoranthene, Chrysene, Dibenzo[*a,h*]anthracene, Fluoranthene, Fluorene, Indeno[1,2,3-*c,d*]pyrene, Naphthalene, Phenanthrene, and Pyrene.

Phenanthrene was always the most frequent PAH detected, whereas dibenzo[*a,h*]anthracene was the only priority PAH not detected at any time. Overall, there was significant inter-person variation in PAH exposures, with volunteers exposed to different PAH congeners and concentrations (Figure 4). Among the seven volunteers who participated in both Spring and Winter, it was possible to quantify both seasonal and within person variation in PAH exposures. Specifically, one PAH (1-methylphenanthrene (58.7 ng/g vs. 28.1 ng/g, *p*-value = 0.02) was significantly higher in Winter than Spring, and two PAHs, fluorine (63.1 ng/g vs. 45.1 ng/g, *p*-value = 0.04) and 2-methylnaphthalene (53.7 ng/g vs. 43.1 ng/g, *p*-value = 0.05) were significantly lower in Winter than Spring. 

Seasonal differences were observed with more priority PAHs detected in the Winter during the colder temperatures. We saw an average change of 3.4 °C between the two seasons, and previous research shows that temperature differentials of less than 4 °C have minimal effect on PAH concentration [64]. Subsequently, temperature was not considered a significant variable, which suggested that other changes between seasons may be responsible for the differing PAH compositions such as increased time spent indoors, heating homes for longer periods of time or using woodstoves which could lead to more exposure from cooking, wood burning stoves and fireplaces, or seasonal changes in fuel compositions. Several factors that were associated with ΣPAH exposure were identified. Specifically, volunteers who reported burning candles indoors, using woodstoves for heat, and coming into contact with gasoline had higher ΣPAH exposures (Table 3). Multiple linear regression models indicated that all three activities were a significant source of ΣPAH exposure after adjustment. While the community was very interested in understanding whether proximity to a known source such as the refineries affected their exposures, the study was not designed to address this specific hypothesis.

### 3.3. Reporting Data

Given that each PAH may come from a different source or have a different toxic potential, we opted to include chemical nomenclature of the 25 detected PAHs measured in the first sampling event (Spring) when reporting results to individuals and the community. However, volunteers reported that the chemical names were unfamiliar and different from anything they knew, and consequently these details made the information harder to remember and more challenging to process. Volunteers pointed out that this type of advanced chemistry was new to the community, and that initial reports should focus on presenting the results as simply as possible using ΣPAH, and then follow up with additional materials that had more detailed information, or include a glossary that defines the chemical terminology so that people could look up unfamiliar words. Parsing out information, as suggested by the volunteers, could provide additional opportunities for the community to learn more about this study and what its results mean to individuals and their community. Additionally, volunteers mentioned that it would be interesting to include a control group or reference population in the study so they could see if the SITC community’s PAH exposures differed from other communities that were not located in close proximity to oil refineries. Furthermore, the lack of an appropriate control group and the continuous operation of the oil refineries during the observation periods hindered the ability to detect any influence of the oil refineries in this study.

### 3.4. Evaluating Impact of Reporting Data

Connecting PAH exposures to specific activities appeared to make volunteers more aware of common sources of PAHs in their community. These data helped maintain awareness of pollution and possible sources, paralleling SITC-led actions to reduce PAH exposures, such as the marine diesel engine and woodstove change out programs, recent remodeling in the casino to reduce exposure to secondhand smoke, and providing seasoned wood to the community. Additionally, the information about sources, coupled with their individual data, allowed people to see how their exposures differed compared to others. This information led some people to change their behaviors to reduce their exposure to sources of PAHs in their home. For instance, one volunteer mentioned that they began buying soy or beeswax candles that have lower emissions and do not burn candles as frequently as they did before the study even though this activity has cultural significance. Other volunteers reported that they no longer used their woodstove as frequently since they participated in the study, citing that it was often used for ambiance rather than heat, whereas others mentioned that the study prompted them to get their woodstove cleaned and serviced before using it the following season. Volunteers also reported that they liked being able to compare their results to others in their community. For those with higher exposures, this information seemed to prompt them to take actions to reduce sources of pollutants in their homes, and for those with lower exposures this information seemed to give a sense of relief because their exposures were not as high as they had originally thought. Since the study could not directly answer the community’s questions about the oil refineries, volunteers still considered it to be an important source of PAH exposures. Despite these limitations, the study appeared to raise awareness about common PAH exposures in the Swinomish community and was perceived to be valuable by the volunteers.

## 4. Discussion

This study utilized novel personal sampling technology to measure personal exposures to polycyclic aromatic hydrocarbon exposures, and followed guidelines for sustainable partnerships [57,65,66,67] where the research questions originated from community concerns about air toxics. The process involves embedding community input into the study design, approach and implementation, interpreting results and directions for research dissemination. This approach is designed to ensure the resultant research addresses the community’s concerns and provides data that can be used by the community to aide in their decision-making. It appeared that this inclusive approach was appreciated by the community, and even though the study was not able to answer all of the community’s questions, especially those questions related to exposures potentially originating from the nearby oil refineries or during a weather inversion, the people who volunteered found the information generated by the study useful and led to increased awareness of sources of pollution that are frequently encountered in their community. Importantly, this community-level information led some people to adopt behaviors that would minimize their exposure to combustion-related pollutants. Additionally, this study provided baseline information on polycyclic aromatic hydrocarbon exposures in the Swinomish community; should another air toxic event occur, staff are now trained to be able to rapidly deploy wristbands in the community in order to collect chemical exposure data which could then be compared to the values measured in this study [68].

Conducting community-engaged science with Native American communities requires tailoring research methodologies to the community’s priorities, recognizing Tribal sovereignty, and acknowledging government-to-government relationships, cultural considerations, historical distrust of academics, and legal considerations regarding data collection and ownership [69,70,71,72,73]. These considerations must be identified and addressed prior to engaging in scientific questioning. Data and Material Sharing and Ownership Agreements (DMSOA) are a useful tool for addressing these issues because they must be negotiated in the beginning of the project, ensuring a mutual understanding of project objectives, anticipated outcomes and Tribal ownership of Tribal information. DMSOAs cover ethical and cultural considerations that often fall outside the scope of Institutional Review Boards and intellectual property laws [60], and they also cover the development of the research plan, the research itself, and the manner in which data are reported back to the community. OSU and SITC have a DMSOA which helps to build trust between the University and the Tribe by ensuring that SITC concerns, data, and materials are legally protected. Another important consideration of community-engaged research is flexibility to ensure the research addresses community concerns and priorities [67,74]. For example, there was more community interest in the personal chemical exposure study following the first deployment in 2016. As a result, the study design was amended to allow increased participation for the second deployment. However, opting for a convenience sample and lack of a control group limited our ability to test specific hypotheses about the contribution of specific PAH sources to personal exposures. Additionally, this study included a relatively small number of volunteers which limited statistical power to detect differences. 

All volunteers opted to receive their results, a trend that has been seen in other community-engaged studies wherein the choice to receive individual results was offered [58]. The TAC also facilitated research translation and ensured that messages were culturally appropriate and did not inadvertently present cultural or spiritual practices in a negative light. For example, initial reports indicated that PAH concentrations were influenced by the use of candles in the home, which is part of spiritual practices. Therefore, the TAC requested that messaging discuss how to reduce emissions from candles instead of reducing candle use. In response to this request, OSU researchers produced an infographic for the community that illustrated how to reduce soot by trimming wicks, using a candle snuffer, and purchasing soy, beeswax or unscented candles [75]. This messaging was further revised to also include other actions individuals could take to reduce exposure to PAHs such as reducing car idling, avoiding smoking, and for Tribal government continue promoting policies that reduce the impacts from industrial emissions (because not all exposures can be reduced by individual behavior changes). 

In conclusion, this community-engaged research leveraged a Tribal-University partnership where state-of-the art exposure assessment was used to document PAH exposures. These data show that all volunteers were exposed to PAHs, although the composition and magnitude of those exposures differed by individual and season. Project personnel used a variety of research translation efforts to raise awareness about these chemical exposures and provide information about exposure reduction strategies. While the limitations of the study hindered the ability to identify sources of air toxics in the community, this activity appeared to raise awareness about ambient and indoor air pollution among the volunteers.

## Figures and Tables

**Table 1:** Description of the people who volunteered to be in the study.

Characteristics	*n* (%)	*n* (%)	*N*
Gender	Male	Female	Total
Spring 2016 (March)	5 (50%)	5 (50%)	10
Winter 2017 (January)	4 (18%)	18 (82%)	22
Participated in both Spring and Winter	3 (43%)	4 (67%)	7
Live/work near point source	Yes	No	Total
Spring 2016 (March)	8 (80%)	2 (20%	10
Winter 2017 (January)	17 (77%)	5 (23%)	22
Participated in both Spring and Winter	5 (71%)	2 (29%)	7

**Table 2:** Descriptive statistics of all 62 PAHs analyzed in the Spring (*n* = 10) and Winter (*n* = 20) deployments. A total of 25 PAHs were detected in Spring and 43 PAHs were detected in Winter.

SPRING—March 2016						WINTER—January 2017				
Variable	# WB detected	Mean	SD	Min.	Max.	# WB detected	Mean	SD	Min.	Max.
Naphthalene *^,†^	10	46.1	40.7	10.5	125	20	31.3	13.9	5.8	61.7
2-methylnaphthalene ^†^	10	65.7	54.9	22.7	201	20	42.9	23.2	18.1	111
1-methylnaphthalene ^†^	10	39.0	29.4	11.9	111	20	28.4	15.0	10.9	77.1
2-ethylnaphthalene ^†^	10	25.1	24.1	6.76	73.6	13	76.5	91.3	6.11	303
2,6-dimethylnaphthalene ^†^	10	100.5	96.4	24	268	12	50.7	46.1	7.24	181
1,6-dimethylnaphthalene ^†^	10	120.3	107.8	31.9	295	18	80.6	73.5	20.1	296
1,4-dimethylnaphthalene ^†^	8	16.7	10.6	4.44	31.5	11	12.3	12.2	2.7	43.9
1,5-dimethylnaphthalene ^†^	10	12.3	9.8	3.03	29.4	11	10.9	8.6	4.74	32.5
1,2-dimethylnaphthalene	0	-	-	-	-	10	26.5	20.0	10.7	76
1,8-dimethylnaphthalene	0	-	-	-	-	0	-	-	-	-
2,6-diethylnaphthalene	2	25.0	23.2	8.6	41.4	0	-	-	-	-
Acenaphthylene *^,†^	3	27.6	17.1	8.2	40.5	8	23.6	13.5	5.52	43.8
Acenaphthene *^,†^	6	33.1	19.0	15.5	69.3	1	52.8	.	52.8	52.8
Fluorene *^,†^	8	63.3	30.1	36.6	121.0	18	48.0	26.3	16.3	109
Dibenzothiophene†	2	11.8	1.6	10.7	12.9	18	19.2	9.9	7.42	44
Phenanthrene *^,†^	10	201.6	114.6	102.0	433.0	20	214.5	108.8	101	466
Anthracene *	0	-	-	-	-	9	58.8	37.1	33.4	150
2-methylphenanthrene ^†^	10	69.8	40.8	30.8	133.0	20	81.1	46.3	29.8	201
2-methylanthracene	0	-	-	-	-	2	12.0	1.6	10.9	13.1
1-methylphenanthrene ^†^	7	31.7	13.4	19.3	53.7	18	56.2	32.6	22.6	143
9-methylanthracene	0	-	-	-	-	0	-	-	-	-
3,6-dimethylphenanthrene	0	-	-	-	-	8	19.7	8.1	7.95	32.7
2,3-dimethylanthracene	0					0	-	-	-	-
Fluoranthene *^,†^	10	59.7	71.6	19.0	204.0	19	66.1	61.9	12.2	267
9,10-dimethylanthracene	0	-	-	-	-	0	-	-	-	-
Pyrene *^,†^	9	63.8	56.2	20.6	187.0	19	61.1	60.6	11.8	281
Retene ^†^	9	89.1	130.6	14.5	428.0	19	84.8	79.2	25	371
Benzo[*a*]fluorene	0	-	-	-	-	8	11.2	6.4	6.34	26.3
Benzo[*b*]fluorene	0	-	-	-	-	1	8.6	.	8.59	8.59
Benzo[*c*]fluorene ^†^	7	12.3	6.7	5.8	23.8	17	15.9	12.6	4.95	57.6
1-methylpyrene ^†^	8	10.0	4.7	4.3	16.2	19	13.8	11.5	3.35	54.8
Benz[*a*]anthracene *	0	-	-	-	-	7	17.5	7.9	6.05	26.4
Cyclopenta[*c,d*]pyrene	0	-	-	-	-	2	10.2	5.1	6.65	13.8
Triphenylene	0	-	-	-	-	6	9.1	3.7	4.59	13.4
Chrysene *^,†^	1	38.1	.	38.1	38.1	6	16.1	9.9	5.34	28.5
6-methylchrysene	0	-	-	-	-	3	12.1	2.3	9.48	13.9
5-methylchrysene	0	-	-	-	-	0	-	-	-	-
Benzo[*b*]fluoranthene *^,†^	2	13.7	4.5	10.5	16.9	4	6.5	4.2	2.49	12.3
7,12-dimethylbenz[*a*]anthracene	0	-	-	-	-	0	-	-	-	-
Benzo[*k*]fluoranthene *^,†^	1	11.5	.	11.5	11.5	4	27.4	28.7	3.3	62.6
Benzo[*j*]fluoranthene *	0	-	-	-	-	4	18.9	17.7	4.56	42.5
Benz[*j*]and[*e*]aceanthrylene	0	-	-	-	-	0	-	-	-	-
Benzo[*e*]pyrene *	0	-	-	-	-	3	17.1	9.7	6.16	24.5
Benzo[*a*]pyrene *	0	-	-	-	-	4	7.7	2.6	5.19	10.2
Indeno[1,2,3-*c,d*]pyrene *	0	-	-	-	-	2	10.3	5.9	6.1	14.4
Dibenzo[*a,h*]anthracene *	0	-	-	-	-	0	-	-	-	-
Benzo[*a*]chrysene	0	-	-	-	-	1	3.2	.	3.19	3.19
Benzo[3,4]perylene *^,†^	1	9.2	.	9.2	9.2	5	5.7	5.8	1.14	12.2
Anthanthrene	0	-	-	-	-	0	-	-	-	-
Naphtho[1,2-*b*]fluoranthene	0	-	-	-	-	1	7.3	-	7.3	7.3
Naphtho[2,3-*j*]fluoranthene	0	-	-	-	-	1	8.6	-	8.55	8.55
Dibenzo[*a,e*]fluoranthene	0	-	-	-	-	0	-	-	-	-
Dibenzo[*a,l*]pyrene	0	-	-	-	-	0	-	-	-	-
Naphtho[2,3-*k*]fluoranthene	0	-	-	-	-	0	-	-	-	-
Naphtho[2,3-*e*]pyrene	0	-	-	-	-	0	-	-	-	-
Dibenzo[*a,e*]pyrene	0	-	-	-	-	0	-	-	-	-
Coronene	0	-	-	-	-	3	2.6	1.6	0.93	4.18
Dibenzo[*e,l*]pyrene	0	-	-	-	-	1	10.0	.	10	10
Naphtho[2,3-*a*]pyrene	0	-	-	-	-	0	-	-	-	-
Benzo[*b*]perylene	0	-	-	-	-	0	-	-	-	-
Dibenzo[*a,i*]pyrene	0	-	-	-	-	0	-	-	-	-
Dibenzo[*a,h*]pyrene	0	-	-	-	-	0	-	-	-	-

**#** WB = the number of wristbands in which the PAH was detected. * indicates priority PAHs as defined by the Agency for Toxic Substance Disease Registry and/or the United States Environmental Protection Agency, † indicates a PAH detected in both Spring and Winter. PAHs below the level of detection are indicated by (-).

**Table 3:** Association between ΣPAH, gender, and activities recorded by volunteers in their daily diaries.

Covariate	*N*	Mean	SD	*p*-Value
Sex				0.5
*Male*	10	1311.5	982.8	
*Female*	20	860.5	424.6	
Candles				0.03
*No*	14	716.6	355.4	
*Yes*	16	1268.3	798.3	
Grilled Food				0.48
*0–4 times per week*	16	1080.5	832.3	
*5–7 times per week*	14	93.2	473.7	
Heat Type				0.05
*Electricity only*	13	782.3	449.1	
*Other (woodstove, wood boiler, natural gas)*	17	1185.6	784.5	
Gasoline Contact				0.03
*0–1 times per week*	19	825.6	450.3	
*2 or more times per week*	11	1330.8	897	
Incense				0.21
*No*	20	1092.3	741.5	
*Yes*	10	847.9	540.5	
Burned Food				0.49
*No*	22	1015.3	705.5	
*Yes*	8	998.6	656.9	
Proximity to a source				0.13
*No*	6	661.9		
*Yes*	23	1070.3		
Season				0.53
*Spring*	10	1024.6	764.5	
*Winter*	20	1004	657.4	

## Supplementary Materials

The following are available online at http://www.mdpi.com/1660-4601/16/3/327/s1, Figure S1: Infographic provided to reduce exposure to candle smoke, Figure S2: Infographic describing various methods of reducing exposure to polycyclic aromatic hydrocarbons.
