# Supplementary material for: A Case Study Describing a Community-Engaged Approach for Evaluating Polycyclic Aromatic Hydrocarbon Exposure in a Native American Community
Source: Int J Environ Res Public Health. 2019 Jan 24;16(3):327. doi: 10.3390/ijerph16030327 (PMC6388274; doi:10.3390/ijerph16030327)
Supplement: Supplementary file 1 [file ijerph-16-00327-s001.pdf]

**Supplemental Figure 1. Infographic provided to reduce exposure to candle smoke.**

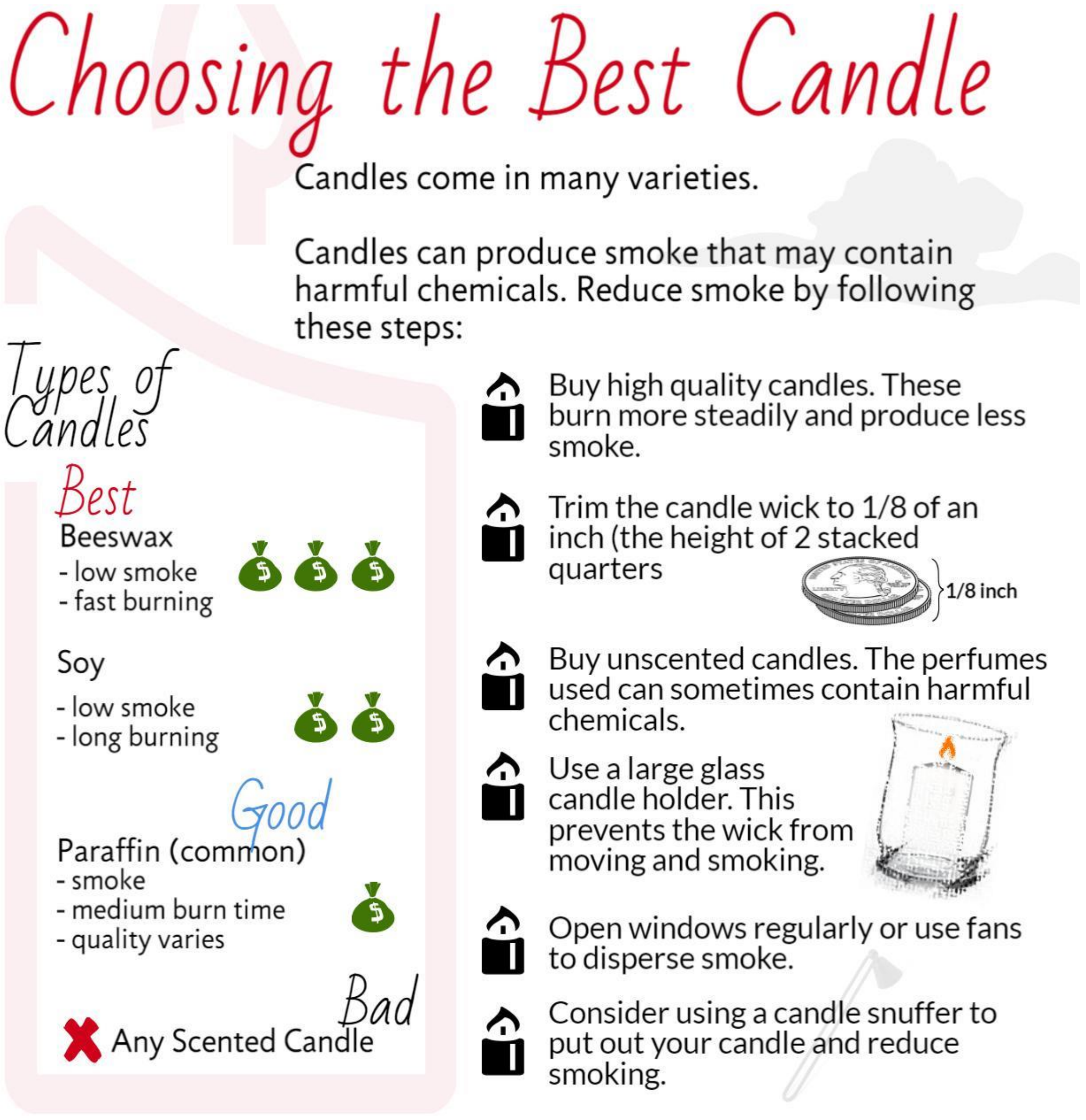

**Supplemental Figure 2. Infographic describing various methods of reducing exposure to polycyclic aromatic hydrocarbons.**

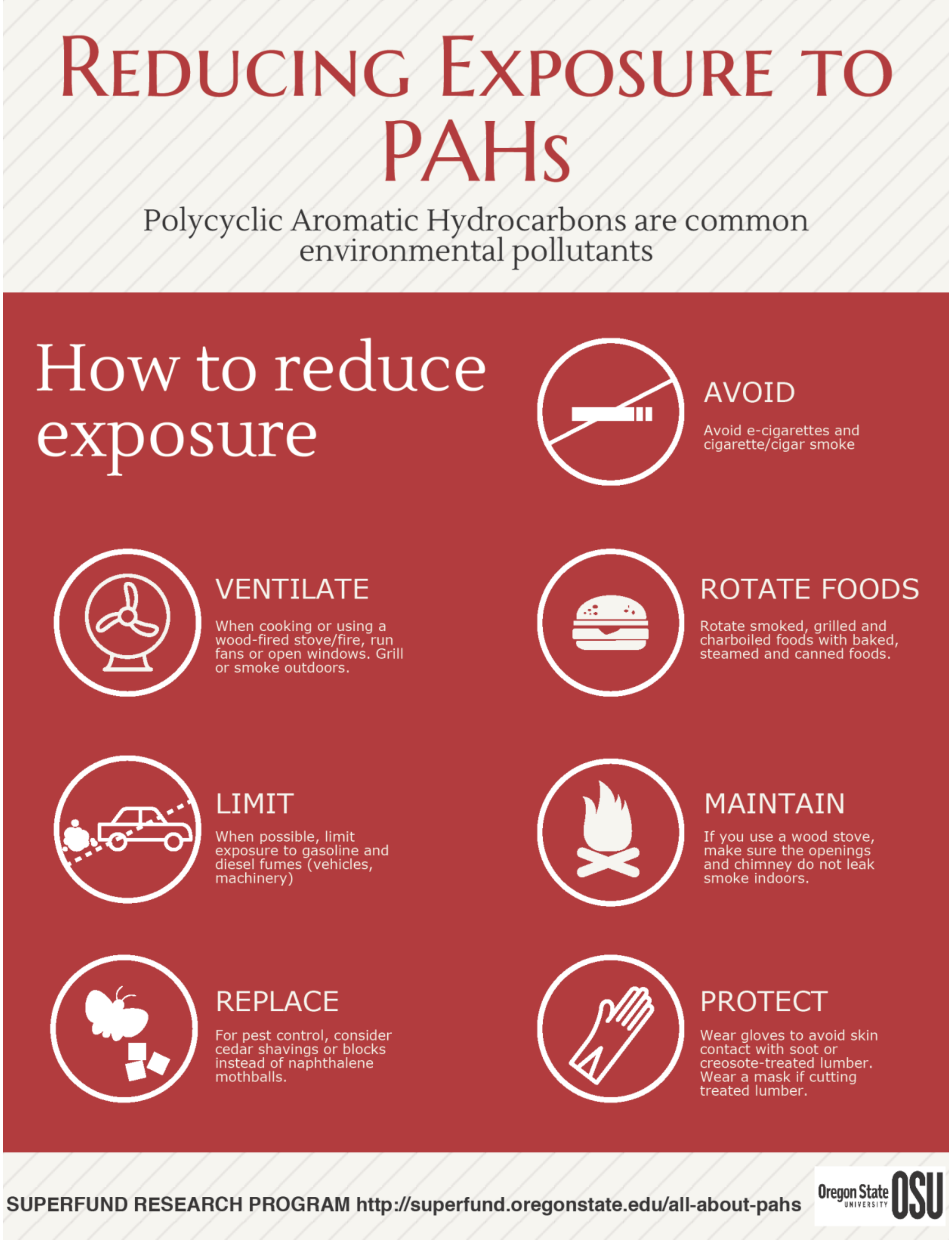

*Could go to International Journal of Environmental Research and Public Health, Environmental Justice, Environmental Health (maybe) or Environmental Health Insights…or other? Preference?*
